# Correction: Playing with fire. Understanding how experiencing a fire in an immersive virtual environment affects prevention behavior

**DOI:** 10.1371/journal.pone.0233123

**Published:** 2020-05-07

**Authors:** The PLOS ONE Staff

## Notice of republication

This article was republished on April 17, 2020, to correct errors in the Funding and Competing interests statements. The publisher apologizes for the errors. Please download this article again to view the correct version.
