# Supplementary material for: Playing with fire. Understanding how experiencing a fire in an immersive virtual environment affects prevention behavior
Source: PLoS One. 2020 Mar 6;15(3):e0229197. doi: 10.1371/journal.pone.0229197 (PMC7059903; doi:10.1371/journal.pone.0229197)
Supplement: S1 File — (DOCX) [file pone.0229197.s001.docx]

**S1 File. Text for INFO group (translated from Dutch).**

**Master the fire!**

With prevention, you simply reduce the chance of damage and limit your damage. Do you have a smoke detector or fire blanket in your house? Then you are well on your way. Below we explain what to do when a pan catches fire when you are cooking.

**What to do when a pan catches fire?**

Pagina-inhoud

A grease fire is caused by overheated fat or fat or oil that runs over the edge of the pan and comes into contact with the stove's fire. A grease fire can, if you do nothing, lead to a house fire within minutes.

A good way to extinguish a grease fire is with a fire blanket. So hang up a fire blanket in the kitchen. Follow the following steps in case of a grease fire:

- Use the fire blanket to extinguish the grease fire by placing the fire blanket over the flame
- Switch off the cooking stove and the exhaust hood.
- Leave the fire blanket on the pan for at least 20 minutes.
- The flame can quickly skip the exhaust hood and the kitchen cabinets. Therefore, clean your exhaust hood monthly.
- Do not walk with the burning pan.
- Never extinguish a grease fire with water, resulting in a burst of flame.
- Caution: Do not use a fire blanket for a deep fryer, as the fire blanket can then be soaked with oil and may catch fire itself.

**What if the flame is too big to extinguish?**

If the flame is bigger than a football, the flame is too big to extinguish with a fire blanket and you have to flee. Pagina-inhoud Working smoke detectors and a good flight plan ensure you can leave your home quickly and safely. In case of fire, the stairs, the hallway and the overflow are sometimes difficult to find or difficult to reach. This is not only due to the fire, but especially by the smoke that is released by the fire.

Therefore, create with your housemates a flight plan. These are agreements on what to do in case of fire. This allows you to leave the house quickly and safely in case of emergency. In case of fire, you have in average 3 minutes to leave the house safely.
