# Supplementary material for: Playing with fire. Understanding how experiencing a fire in an immersive virtual environment affects prevention behavior
Source: PLoS One. 2020 Mar 6;15(3):e0229197. doi: 10.1371/journal.pone.0229197 (PMC7059903; doi:10.1371/journal.pone.0229197)
Supplement: S2 File — (DOCX) [file pone.0229197.s002.docx]

**S2 File. Description of scenarios and feedback in IVE (translated from Dutch).**

| **Scenario** | **Short description** | **Full description** |
| --- | --- | --- |
| 1 | Fire blanket | Players goes to the kitchen, extinguishes the fire with the fire blanket within 30 seconds. The fire dies. Player sees the fire blanket over the pan. |
| 2 | Bucket of water, escape with child, on time | Player goes to the kitchen, extinguishes the fire with water within 30 seconds. Player sees a burst of flame. Player escapes, goes upstairs to pick up the toddler, and safely leaves house within 105 seconds. |
| 3 | Bucket of water, escape without child, on time | Player goes to the kitchen, extinguishes the fire with water within 30 seconds. Player sees a burst of flame. Player leaves the house within 105 seconds. |
| 4 | Bucket of water, escape, too late | Player goes to the kitchen, extinguishes the fire with water within 30 seconds. Player sees a burst of flame. Player does not leave house within 105 seconds. |
| 5 | Escape with child, on time | Player goes upstairs to pick-up toddler, and leaves house within 105 seconds. |
| 6 | Escape without child, on time | Player leaves the house within 105 seconds. |
| 7 | Escape, too late | Player does not leave the house within 105 seconds. |

After each scenario the following textual feedback was given:

**Scenario 1: fire blanket**

Perfect! You have extinguished the fire on time with a fire blanket which prevented a lot of damage.

- For a grease fire, use a fire blanket to extinguish the flame.
- Switch off the cooking stove and the exhaust hood.
- Leave the fire blanket on the pan for at least 20 minutes.
- The flame can quickly skip the exhaust hood and the kitchen cabinets. Therefore, clean your exhaust hood monthly.
- Do not walk with the burning pan.
- Caution: Do not use a fire blanket for a deep fryer, as the fire blanket can then be soaked with oil and may catch fire itself.

**Scenario 2: Bucket of water, escape with child, on time**

Fine! You and your family left the house on time. However, the fire caused a lot of damage to your house and belongings.

Make a flight plan together with your housemates. These are agreements about what to do in the case of a fire, so that you can leave your house quickly and safely. In case of a fire, you have an average of 3 minutes to leave your house safely.

By throwing water on a grease fire, a burst of flame is created. The water evaporates and takes oil droplets which then catch fire. A fire blanket is a good way to extinguish a grease fire. Try to extinguish the grease fire within 30 seconds.

Give it a try again!

**Scenario 3: Bucket of water, escape without child, on time**

Fine! You have left the house on time. However, there is still a family member left behind.

You can prevent this by making a flight plan. These are agreements you make with your housemates about what to do in the event of a fire, so that you can leave your home quickly and safely. In case of a fire, you have an average of 3 minutes to leave your home safely.

By throwing water on a grease fire, a burst of flame is created. The water evaporates and takes oil droplets which then catch fire. A fire blanket is a good way to extinguish a grease fire. Try to extinguish the grease fire within 30 seconds.

Give it a try again!

**Scenario 4: Bucket of water, escape, too late**

Unfortunately! You did not leave the house on time.

In case of a fire, you have an average of 3 minutes to safely leave your home. Make a flight plan together with your housemates. These are agreements about what to do in case of a fire, so that you can leave your home quickly and safely.

By throwing water on a grease fire, a burst of flame is created. The water evaporates and takes oil droplets which then catch fire. A fire blanket is a good way to extinguish a grease fire. Try to extinguish the grease fire within 30 seconds.

Give it a try again!

**Scenario 5: escape with child on time**

Very well! You and your family left the house on time. However, the fire caused a lot of damage to your house and belongings.

Make a flight plan together with your housemates. These are agreements about what to do in the event of a fire, so that you can leave your home quickly and safely. In the event of a fire, you have an average of 3 minutes to safely leave your home.

A fire blanket is a good way to extinguish a flame in the pan. Try to extinguish the grease fire within 30 seconds.

Give it a try again!

**Scenario 6: escape without child, on time**

Fine! You have left the house on time. However, there is still a family member left behind.

You can prevent this by making a flight plan. These are agreements you make with your housemates about what to do in case of a fire, so that you can leave your home quickly and safely. In case of a fire, you have an average of 3 minutes to safely leave your home.

A fire blanket is a good way to extinguish a grease fire. Try to extinguish the grease fire within 30 seconds.

Give it a try again!

**Scenario 7: escape too late**

Unfortunately! You did not leave the house on time.

In case of a fire, you have an average of 3 minutes to safely leave your home. Make a flight plan together with your housemates. These are agreements about what to do in case of a fire, so that you can leave your home quickly and safely.

A fire blanket is a good way to extinguish a flame in the pan. Try to extinguish the grease fire within 30 seconds.

Give it a try again!
