# Supplementary material for: Playing with fire. Understanding how experiencing a fire in an immersive virtual environment affects prevention behavior
Source: PLoS One. 2020 Mar 6;15(3):e0229197. doi: 10.1371/journal.pone.0229197 (PMC7059903; doi:10.1371/journal.pone.0229197)
Supplement: S3 File — (DOCX) [file pone.0229197.s003.docx]

**S3 File. Knowledge scale (translated from Dutch).**

1. What happens when you use water to extinguish a grease fire? [open answer]

*Correct answer: a burst of flame arises*

1. What happens when you use a fire blanket to extinguish the grease fire? [open answer]

*Correct answer: the fire extinguishes*

1. Is a fire blanket suitable to extinguish a deep fryer fire?

- Yes
- No
- I do not know

*Correct answer: no*

Indicate if it is a good idea, in case of a grease fire, to:

[yes; no; I do not know]

1. Turn off the cooking stove
2. Turn off the exhaust hood
3. Take the burning pan outside
4. If you have extinguished the flame, check immediately if the flame is out

*Correct answers: [4] yes; [5] yes; [6] no; [7] no*

1. How often does the exhaust hood have to be cleaned to prevent fire?

- Once a week
- Once a month
- Once every three months
- Once a half year
- Once a year

*Correct answer: once a month*

1. On average, how many minutes does someone have to safely leave their home in case of fire?

Give an answer between 0-30 minutes [open answer]

*Correct answer: 3 minutes*
