# Supplementary material for: Playing with fire. Understanding how experiencing a fire in an immersive virtual environment affects prevention behavior
Source: PLoS One. 2020 Mar 6;15(3):e0229197. doi: 10.1371/journal.pone.0229197 (PMC7059903; doi:10.1371/journal.pone.0229197)
Supplement: S4 File — (DOCX) [file pone.0229197.s004.docx]

**S4 File. Additional results of Confirmatory Factor Analysis**

**Table A.** **Factor loadingsand Cronbach’s Alpha (α) when performing CFA with all factors together of the original scales (one time with ISLOC and ESLOC, and one time with SLOC)**

| Original scale  (with ISLOC and ESLOC) | Items | Factor loading  (stdYX) | Original scale (with SLOC) | Items | Factor loading  (stdYX) |
| --- | --- | --- | --- | --- | --- |
| ISLOC | ISLOC1 | .534 | SLOC | ISLOC1 | .498 |
|  | ISLOC5 | .723 |  | ESLOC2 | -.326 |
|  | ISLOC7 | .687 |  | ESLOC3 | .024 |
|  | ISLOC9 | .516 |  | ESLOC4 | -.528 |
|  | ISLOC10 | .784 |  | ISLOC5 | .689 |
|  | ISLOC11 | .151 |  | ESLOC6 | -.336 |
|  | *α* | *.583* |  | ISLOC7 | .642 |
|  |  |  |  | ESLOC8 | -.779 |
| ESLOC | ESLOC2 | .354 |  | ISLOC9 | .493 |
|  | ESLOC3 | -.007 |  | ISLOC10 | .742 |
|  | ESLOC4 | .570 |  | ISLOC11 | .142 |
|  | ESLOC6 | .367 |  | ESLOC12 | -.778 |
|  | ESLOC8 | .831 |  |  |  |
|  | ESLOC12 | .828 |  |  |  |
|  | *α* | *.587* |  | *α* | *.658* |
| Vulnerability | VUL10 | .618 | Vulnerability | VUL10 | .620 |
|  | VUL11 | .653 |  | VUL11 | .651 |
|  | VUL12 | .971 |  | VUL12 | .972 |
|  | *α* | *.745* |  | *α* | *.745* |
| Severity | SEV13 | .827 | Severity | SEV13 | .827 |
|  | SEV14 | .826 |  | SEV14 | .825 |
|  | SEV15 | .885 |  | SEV15 | .886 |
|  | *α* | *.752* |  | *α* | *.752* |
| Self-efficacy | SELF1 | .798 | Self-efficacy | SELF1 | .798 |
|  | SELF2 | .827 |  | SELF2 | .827 |
|  | SELF3 | .668 |  | SELF3 | .668 |
|  | SELF4 | .778 |  | SELF4 | .778 |
|  | SELF5 | .729 |  | SELF5 | .729 |
|  | SELF6 | .832 |  | SELF6 | .832 |
|  | SELF7 | .760 |  | SELF7 | .760 |
|  | SELF8 | .691 |  | SELF8 | .691 |
|  | SELF9 | .682 |  | SELF9 | .682 |
|  | SELF10 | .694 |  | SELF10 | .694 |
|  | *α* | *.863* |  | *α* | *.863* |

**Table B.** **Model fit scores of original scales, when performing CFA on individual scales**

| Goodness of fit | | Target values | ESLOC | ISLOC | SLOC | Vulnera-  bility* | Severity* | Self-efficacy |
| --- | --- | --- | --- | --- | --- | --- | --- | --- |
| χ^2^ |  | | 19.025 | 58.007 | 237.912 | .000 | .000 | 672.567 |
| df |  | | 9 | 9 | 54 | 0 | 0 | 35 |
| *p* | >.05 | | .025 | <.001 | <.001 | <.001 | <.001 | <.001 |
| *RMSEA* | <.06 | | .068 | .150 | .119 | .000 | .000 | .274 |
| 90% *CI* | <.10 | | .023-.111 | .115-.188 | .103-.134 | .00-.00 | .00-.00 | .256-.293 |
| *CFI* | >.95 | | .982 | .918 | .894 | 1.00 | 1.00 | .846 |
| *TLI* | >.95 | | .969 | .863 | .870 | 1.00 | 1.00 | .801 |

*Note. model is “just identified” (*df*= 0)

**Table C.** **Model fit scores of modified scales, when performing CFA on individual scales**

| Goodness of fit | Target values | ESLOC* | ISLOC* | SLOC | Vulnera-bility* | Severity* | Self-efficacy |
| --- | --- | --- | --- | --- | --- | --- | --- |
| χ^2^ |  | .000 | .000 | 24.813 | .000 | .000 | 192.345 |
| df |  | 0 | 0 | 5 | 0 | 0 | 5 |
| *p* | >.05 | <.001 | <.001 | <.001 | <.001 | <.001 | <.001 |
| *RMSEA* | <.06 | .000 | .000 | .128 | .000 | .000 | .393 |
| 90% *CI* | <.10 | .00-.00 | .00-.00 | .081--.180 | .00-.00 | .00-.00 | .347-.442 |
| *CFI* | >.95 | 1.000 | 1.000 | .984 | 1.00 | 1.00 | .935 |
| *TLI* | >.95 | 1.000 | 1.000 | .969 | 1.00 | 1.00 | .869 |

*Note. model is “just identified” (*df*=0)

**Table D.** **Model fit scores of original scales, when performing CFA on all scales together**

| Goodness of fit | Target values | With SLOC | With ISLOC and ESLOC |
| --- | --- | --- | --- |
| χ^2^ |  | 1087.979 | 1080.666 |
| df |  | 344 | 340 |
| *p* | >.05 | <.001 | <.001 |
| *RMSEA* | <.06 | .095 | .095 |
| 90% *CI* | <.10 | .088-.101 | .089-.101 |
| *CFI* | >.95 | .874 | .875 |
| *TLI* | >.95 | .862 | .861 |

**Table E.** **Model fit scores of modified scales, when performing CFA on all scales together**

| Goodness of fit | Target values | With SLOC | With ISLOC and ESLOC |
| --- | --- | --- | --- |
| χ^2^ |  | 478.448 | 521.170 |
| df |  | 98 | 109 |
| *p* | >.05 | <.001 | <.001 |
| *RMSEA* | <.06 | .127 | .125 |
| 90% *CI* | <.10 | .115-.138 | .114 -.136 |
| *CFI* | >.95 | .914 | .906 |
| *TLI* | >.95 | .895 | .882 |

**Table F.** **R-squared estimates (**$\mathbf{R}^{\mathbf{2}}\mathbf{)}$**and Average Variance Extracted (AVE) when performing CFA with all factors together and with the individual scales separately (with SLOC instead of ISLOC and ESLOC)**

|  |  | CFA with all factors | | CFA with individual scales | |
| --- | --- | --- | --- | --- | --- |
|  |  | Original scale | Modified scale | Original scale | Modified scale |
|  |  | $R^{2}$ | $R^{2}$ | $R^{2}$ | $R^{2}$ |
| SLOC | ISLOC1 | .285 |  | .191 |  |
|  | ESLOC2 | .126 |  | .112 |  |
|  | ESLOC3 | 0 |  | 0 |  |
|  | ESLOC4 | .304 |  | .262 |  |
|  | ISLOC5 | .523 | .427 | .408 | .351 |
|  | ESLOC6 | .135 |  | .125 |  |
|  | ISLOC7 | .472 | .437 | .417 | .448 |
|  | ESLOC8 | .69 | .604 | .576 | .549 |
|  | ISLOC9 | .266 |  | .250 |  |
|  | ISLOC10 | .615 | .538 | .611 | .593 |
|  | ISLOC11 | .023 |  | .030 |  |
|  | ESLOC12 | .686 | .634 | .654 | .673 |
|  | *AVE* | *.304* | *.528* | *.303* | *.523* |
| Vulnerability | VUL10 | .385 | .325 | .369 | .369 |
|  | VUL11 | .423 | .435 | .583 | .583 |
|  | VUL12 | .945 | *undefined* | .754 | .754 |
|  | *AVE* | *.584* |  | *.569* | *.569* |
| Severity | SEV13 | .683 | .687 | .799 | .799 |
|  | SEV14 | .681 | .723 | .954 | .954 |
|  | SEV15 | .786 | .705 | .293 | .293 |
|  | *AVE* | *.717* | *.705* | *.682* | *.682* |
| Self-efficacy | SELF1 | .637 | .696 | .627 | .695 |
|  | SELF2 | .683 | .718 | .666 | .702 |
|  | SELF3 | .446 |  | .469 |  |
|  | SELF4 | .605 | .612 | .581 | .583 |
|  | SELF5 | .531 | .596 | .532 | .609 |
|  | SELF6 | .692 | .789 | .704 | .820 |
|  | SELF7 | .578 |  | .576 |  |
|  | SELF8 | .477 |  | .481 |  |
|  | SELF9 | .466 |  | .485 |  |
|  | SELF10 | .482 |  | .488 |  |
|  | *AVE* | *.560* | *.682* | *.561* | .682 |

**Table G.** **R-squared estimates (**$\mathbf{R}^{\mathbf{2}}\mathbf{)}$**and Average Variance Extracted (AVE) when performing CFA with all factors together and with the individual scales separately (with ISLOC and ESLOC instead of SLOC)**

|  |  | CFA with all factors | | CFA with individual scales | |
| --- | --- | --- | --- | --- | --- |
|  |  | Original scale | Reduced scale | Original scale | Reduced scale |
|  |  | $R^{2}$ | $R^{2}$ | $R^{2}$ | $R^{2}$ |
| ISLOC | ISLOC1 | .285 |  | .263 |  |
|  | ISLOC5 | .523 | .500 | .444 | .238 |
|  | ISLOC7 | .472 |  | .324 |  |
|  | ISLOC9 | .266 | .325 | .365 | .329 |
|  | ISLOC10 | .615 | .734 | .789 | *undefined* |
|  | ISLOC11 | .023 |  | .034 |  |
|  | *AVE* | *.364* | .413 | *.369* |  |
| ESLOC | ESLOC2 | .126 |  | .180 |  |
|  | ESLOC3 | 0 |  | .027 |  |
|  | ESLOC4 | .423 | .339 | .384 | .381 |
|  | ESLOC6 | .135 |  | .196 |  |
|  | ESLOC8 | .690 | .685 | .667 | .677 |
|  | ESLOC12 | .686 | .624 | .588 | .595 |
|  | *AVE* | *.343* | *.549* | *.340* | *.551* |
