# Supplementary material for: Playing with fire. Understanding how experiencing a fire in an immersive virtual environment affects prevention behavior
Source: PLoS One. 2020 Mar 6;15(3):e0229197. doi: 10.1371/journal.pone.0229197 (PMC7059903; doi:10.1371/journal.pone.0229197)
Supplement: S1 Table — (DOCX) [file pone.0229197.s005.docx]

**S1 Table. Actions people took in the IVE fire game, during the first and second game play.**

| **Scenario** | **Action** | **First game** | **Second game** |
| --- | --- | --- | --- |
| 1 | Fire blanket | 56.1% | 43.1% |
| 2 | Bucket of water, escape with child, on time | 8.1% | 13.8% |
| 3 | Bucket of water, escape without child, on time | .8% | 1.6% |
| 4 | Bucket of water, escape, too late | 3.3% | 5.7% |
| 5 | Escape with child, on time | 20.3% | 28.5% |
| 6 | Escape without child, on time | 7.3% | .8% |
| 7 | Escape, too late | 4.1% | 4.9% |
|  | Did not play |  | 1.6% |

Note. N=123
